# PRL-3 suppresses c-Fos and integrin α2 expression in ovarian cancer cells

**DOI:** 10.1186/1471-2407-13-80

**Published:** 2013-02-18

**Authors:** Hao Liu, Abdul Qader Omer Al-aidaroos, Haihe Wang, Ke Guo, Jie Li, Hua Fei Zhang, Qi Zeng

**Affiliations:** 1 MOE key laboratory of Industrial Fermentation Microbiology, College of Biotechnology, Tianjin University of Science and Technology, Tianjin, 300457, People’s Republic of China; 2 Institute of Molecular and Cell Biology, A*STAR (Agency for Science, Technology and Research), 61 Biopolis Drive, Proteos, Singapore, 138673, Republic of Singapore; 3 Department of Biochemistry, Zhongshan School of Medicine, Sun Yat-Sen University, 74 Zhongshan Road II, Guangzhou, Guangdong, 510080, People’s Republic of China; 4 Department of Biochemistry, Yong Loo Lin School of Medicine, National University of Singapore, Singapore, 119260, Republic of Singapore

**Keywords:** PRL-3 phosphatase, Cancer metastasis, Integrin α2, c-fos transcription factor, Adhesion molecule, Cell migration

## Abstract

**Background:**

Phosphatase of regenerating liver-3 (PRL-3), a protein tyrosine phosphatase, is highly expressed in multiple human cancers and strongly implicated in tumor progression and cancer metastasis. However, the mechanisms by which PRL-3 promotes cancer cell migration, invasion, and metastasis are not very well understood. In this study, we investigated the contribution and molecular mechanisms of PRL-3 in ovarian cancer progression.

**Methods:**

PRL-3 protein expression was detected on ovarian cancer tissue microarrays using immunohistochemistry. Stable PRL-3 depleted cell lines were generated using short hairpin RNA (shRNA) constructs. The migration and invasion potential of these cells were analyzed using Transwell and Matrigel assays, respectively. Immunoblotting and immunofluorescence were used to detect protein levels and distribution in PRL-3-ablated cells and the control cells. Cell morphology was observed with hematoxylin-eosin staining and transmission electron microscopy. Finally, PRL-3-ablated and control cells were injected into nude mice for xenograft tumorigenicity assays.

**Results:**

Elevated PRL-3 expression was detected in 19% (26 out of 135) of human ovarian cancer patient samples, but not in normal ovary tissues (0 out of 14). Stable depletion of PRL-3 in A2780 ovarian cancer cells resulted in decreased migration ability and invasion activity compared with control parental A2780 cells. In addition, PRL-3-ablated cells also exhibited flattened morphology and extended lamellipodia. To address the possible molecular basis for the altered phenotypes associated with PRL-3 down-regulation, we assessed the expression profiles of various proteins involved in cell-matrix adhesion. Depletion of PRL-3 dramatically enhanced both RNA and protein levels of the cell surface receptor integrin α2, but not its heterologous binding partner integrin β1. Inhibition of PRL-3 also correlated with elevated expression and phosphorylation of paxillin. A pronounced increase in the expression and activation of c-fos, a transcriptional activator of integrin α2, was observed in these PRL-3 knock-down cells. Moreover, forced expression of EGFP-PRL-3 resulted in the suppression of both integrin α2 and c-fos expression in A2780 cells. Significantly, using a xenograft tumor model, we observed a greatly reduced tumorigenicity of A2780 PRL-3 knock-down cells *in vivo.*

**Conclusions:**

These results suggest that PRL-3 plays a critical role in ovarian cancer tumorigenicity and maintaining the malignant phenotype. PRL-3 may inhibit c-fos transcriptional regulation of integrin α2 signaling. Our results strongly support a role for PRL-3 as a promising therapeutic target and potential early biomarker in ovarian cancer progression.

## Background

Metastasis – the spread of cells from the primary neoplasm to distant organs and their relentless growth – is the cause of 90% of human cancer mortality [1]. The process of metastasis consists of a long series of sequential, interrelated steps, and their cellular, genetic and biochemical determinants remain incompletely understood. A critical aspect of metastatic behavior involves adhesive interactions of tumor cells with other cells or with the extracellular matrix [2]. Several classes of proteins involved in the tethering of cells to their surroundings in a tissue are altered in cells possessing metastatic capabilities. One of the most widely observed cell surface changes in cancer cells is in integrin expression. Integrins comprise of a family of heterodimeric cell adhesion receptors which mediate a wide variety of cell-cell and cell-matrix interactions that lead to cell migration, proliferation, differentiation and survival [3,4]. For instance, the enhanced metastatic potential of B16a melanoma cells is mediated by increased expression of αIIbβ3 receptors at the transcriptional level [5]. In contrast, decreasing the expression of α2β1 integrin in breast carcinoma cells results in dramatic morphological alterations, whilst re-expression of α2β1 integrin restores the ability to differentiate and markedly reduces the in vivo tumorigenicity of these cells [6]. More recently, the α2β1 heterodimer has also been shown to negatively regulate metastasis of murine and human cancers [7].

Accumulating evidence indicates that the dysregulated expression of the phosphatase of regenerating livers (PRLs) is linked to cancer cell proliferation, migration, invasion and metastasis [8]. Global gene expression profiles reveal that PRL-3 was expressed at higher levels in metastatic colorectal carcinomas but at lower levels in non-metastatic tumors and normal colorectal epithelium [9]. In addition to colorectal carcinomas, high PRL-3 expression is also frequently detected during the development or advancement of breast, gastric, ovarian, and liver carcinomas [10]. Consistent with a role of high-level expression of PRL-3 in metastasis, we demonstrated that ectopic expression of PRL-3 in Chinese hamster ovary cells enhanced motility, invasive activity and induced metastatic tumor formation in mice [11], suggesting that elevated expression of PRL-3 phosphatase may be a key contributor to the metastasis of the transformed cells. Indeed, transient down-regulation of PRL-3 expression with small interfering RNA (siRNA) in DLD-1 colorectal cancer cells abrogated motility *in vitro* and hepatic colonization *in vivo*[12], and down-regulation of PRL-3 in breast cancer cells [13], melanoma cells [14], and gastric cancer cells [15] also consistently reduced motility and metastasis. In ovarian cancers, PRL-3 expression levels correlate with disease progression, being higher in advanced (stage III) than in early (stage I) tumors [16]. Although depletion of PRL-3 using siRNA impaired the proliferation of ovarian cancer cells [16], a role for PRL-3 in the migration or invasion of ovarian cancers has not been reported.

Here, we further characterized the expression and role of PRL-3 in human ovarian cancers. We detected PRL-3 expression specifically in cancer tissues, but not normal tissues, of the ovary. Importantly, depletion of PRL-3 resulted in increased cell spreading, decreased motility and invasiveness, as well as reduced tumorigenicity of A2780 ovarian cancer cells. These observations were concomitant with a profound increase in integrin α2 expression, as well its transcriptional regulator, c-Fos. Our results propose a role for PRL-3 in the early progression of ovarian cancers, and highlight its potential utility as an ovarian cancer early biomarker.

## Materials and methods

### Cell lines and cell culture

Human ovarian cancer cell line A2780 was purchased from the American Type Culture Collection (ATCC, VA) and routinely maintained in RPMI 1640 (Invitrogen) supplemented with heat-inactivated 10% (v/v) fetal bovine serum (Invitrogen) and 1% antibiotic-antimycotic (PAA Laboratories) at 37°C in a humidified atmosphere of 5% CO2.

### Tissue samples and IHC analysis

The ovary cancer tissue arrays (Ovary Cancer TMA, Catalog ID: CC11-11-005 and CC11-10-001) were purchased from Cybrdi, Inc. (Rockville, Maryland). We used EnVisionTM Systems K 1395 (Dako) to perform IHC analysis.

### Generation of stable cell lines

For PRL-3 knockdown, 8 shRNA constructs against human PRL-3 purchased from OriGene (catalogue #TR320652) and SABiosciences (catalogue #KH09221) were used to knock down PRL-3 in A2780 cells. Transient knocking down assays suggested that the constructs containing insert sequences: 5’-CGGCAAGGTAGTGGAAGACTGGCTGAGCC-3’ (PRL-3 KD-22) and 5’-TTCTCGGCACCTTAAATTATT-3’ (PRL-3 KD-S3) were most efficient (data not shown). These two constructs were subsequently used to establish PRL-3 suppressed stable cell lines. In brief, the above two PRL-3 specific constructs and one control vector were transfected into A2780 cells using using Lipofectamine 2000 (Invitrogen). The cells were cultured in RPMI 1640 supplemented with 10% FBS and selected in 1 mg/ml of neomycin for 14 days. Thereafter, individual colonies were picked and tested for PRL-3 expression level by semi-quantitative RT-PCR and immunoblotting. For generation of cells overexpressing EGFP-PRL-3, A2780 cells were transfected with EGFP-PRL-3 plasmid [17] using Lipofectamine 2000 (Invitrogen). Two days after transfection, 1 mg/ml of neomycin was added to the culture dishes, and drug-resistant cells were allowed to grow for 21 days. Individual neomycin stable colonies were picked and examined for EGFP fluorescence using confocal microscopy.

### Semi quantitative RT-PCR

Total RNAs were isolated using TRIzol reagent (Invitrogen) according to the manufacturer’s instructions. The purity and concentration of RNA was determined spectrophotometrically (ND-1000, Nanodrop Technologies), and quality assessed using the Agilent Bioanalyzer 2100 (Agilent Technologies Inc.). Reverse transcription-PCR was performed with Super-Script one-step RT-PCR kit (Invitrogen) according to the manufacturer’s instructions. Equal amounts of RNA (200 ng) were used as templates in each reaction. The primer sets used for PCR amplification are listed in Additional file 1: Table S1. PCR products were electrophoresed on a 1.5% agarose gel and visualized using GelRed staining (Biotium).

### Western blot analysis

Cells at 80% confluence were washed thrice with cold PBS and lysed in 50 mmol/L Tris–HCl (pH 7.4), 250 mmol/L NaCl, 0.1% Nonidet NP40, 5 mmol/L EDTA, 50 mmol/L NaF in the presence of aprotinin, leupeptine, and phenylmethylsulfonyl fluoride as protease inhibitors for 30 minutes on ice. Cell lysates were then clarified by centrifugation (14,000 rpm) at 4°C for 15min. Protein concentration of the lysates was determined using a Bradford assay kit (Bio-Rad). Following SDS-PAGE electrophoresis, proteins were transferred onto nitrocellulose membranes and probed with various antibodies. Antibodies against integrin α2, integrin αV, integrin β1, FAK, phospho-FAK (pY397), Erk1/2, phospho-Erk1/2 (pT202/pY204), JNK, phospho-JNK (pT183/pY185), p38, phospho-p38 (pT180/pY182) were from BD Biosciences. Antibodies against paxillin and phospho-paxillin (pY195), phospho-paxillin (pY141), and phospho-paxillin (pS178) were from ECM Biosciences. Antibodies agasint phospho-paxillin (pY118), phospho-FAK (pY925), phospho-FAK (pY576/577), c-fos and c-Jun were from Cell Signaling Technology. Antibodies against phospho-paxillin (pY31) and Sp1 were from Abcam. PRL-3 monoclonal antibody was generated in our lab as previously described [18].

### Hematoxylin-eosin staining

Exponentially growing cells grown on cover glasses were fixed in 4% paraformaldehyde for 20 min, briefly rinsed in PBS several times, followed by washing under running water for 5 min. The coverglasses were stained in Haematoxylin solution for 5 min and washed under running water until excess stain was removed. The slides were dipped in acid-ethanol (1% concentrated hydrochloric acid (v/v), 70% ethanol (v/v)) and washed again under running water for another 5 min. The slides were then stained in Eosin-ethanol (1% Eosin Y (w/v) in 80% ethanol (v/v)) for 3 min, subjected to sequential dehydration, and mounted for analysis under an Axioplan upright microscope (Carl Zeiss AG) equipped with a SPOT Insight color camera (SPOT Imaging).

### Transmission electron microscopy (TEM)

A standard protocol was followed for transmission electron microscopy. Briefly, samples were fixed with glutaraldehyde (2.5%, v/v) in 0.1 M phosphate buffer (pH 7.4) at 37°C. After fixation, samples were placed in 2% osmium tetroxide in 0.1 M sodium cacodylate buffer (pH 7.4), dehydrated in a graded series of ethyl alcohol, and viewed with a JEM1010 transmission electron microscope (Jeol, Tokyo, Japan) at 100 kV. Light microscopic examination was performed using a Leica DMLB microscope. Images were captured with an Optronics DEI-750T CCD camera (Muskogee, OK) and Leica Qwin software.

### Immunofluorescence

Cells growing on coverslips were fixed with 4% paraformaldehyde at room temperature for 10 min, cells were washed twice with PBS and permeabilized with 0.1% Triton X-100 for 5 min. After blocking with 1% bovine serum albumin (Sigma-Aldrich), cells were incubated with the indicated antibodies for 2 h. After washing thrice with PBS, a corresponding fluorochrome-labeled secondary antibody was added and incubated for 1 h. Cells were then rinsed thrice with PBS and To-pro-3 iodide was used to stain DNA. Fluorescence images were captured and analyzed using an LSM510 confocal microscope (Carl Zeiss AG).

### Cell migration and invasion assays

Cell migration assay was performed using Transwell inserts (6.5 mm diameter; 8 μM pore size polycarbonate membrane) obtained from Corning Glass (Cambridge, MA). In brief, after overnight serum starvation, 1 × 10^5^ cells in 0.5 mL serum-free RPMI 1640 medium were placed in the upper chamber, and the lower chamber was loaded with 0.8 mL medium containing 10% FBS. After 24 hours incubation at 37°C with 5% CO2, cells that migrated to the lower chamber were fixed with 4% paraformaldehyde, stained with a solution containing 0.5% crystal violet and 2% ethanol in 100 mM borate buffer, and then counted with hematoxylin under a light microscope. For cell invasion assays, Matrigel (BD Biosciences) was used to coat the upper surface of the chambers according to the manufacturer’s instructions, and the coated inserts subsequently used in a similar manner to the above-described migration assay.

### Mice xenograft tumorigenicity assay

1 × 10^6^ A2780 Vector (control) or A2780 PRL-3 KD-22 cells were injected respectively into the left or right side of the hip areas of 8-week old nude mice (Jackson Labs) to examine the tumorigenicity of the cells *in vivo*. The mice and tumors were monitored during the whole course of experiments. The experiment was terminated after 5 weeks, and mice were photographed with a digital camera (Nikon). All animal studies were approved by the Institutional Animal Care and Use Committee (IACUC) and were carried out under the policies of Institute of Molecular and Cell Biology’s Review Board (IRB), Singapore.

### Microarray dataset analysis

The GSE9891 dataset consists of 285 primary ovarian cancer specimens assayed on the Affymetrix HG-U133 Plus 2.0 platform [19]. The dataset was obtained from the Gene Expression Omnibus (GEO) repository in preprocessed soft format. The targeting probesets used were ‘206574_s_at’ and ‘209695_at’ (for PRL-3; PTP4A3) and ‘205032_at’ and ‘227314_at’ (for integrin α2; ITGA2). The average expression levels of each gene’s probesets were used for statistical analysis. The association between mRNA expression of PRL-3 and integrin α2 was analyzed using Spearman’s rank test using the SPSS 15.0 software package (IBM), and *p* values < 0.05 were considered statistically significant.

### Ethical approval

The use of all human tissue samples were approved by the Institutional Review Board (IRB) of the Institute of Molecular and Cell Biology, Singapore.

## Results

### PRL-3 is upregulated in human ovarian cancers

**Figure 1:**
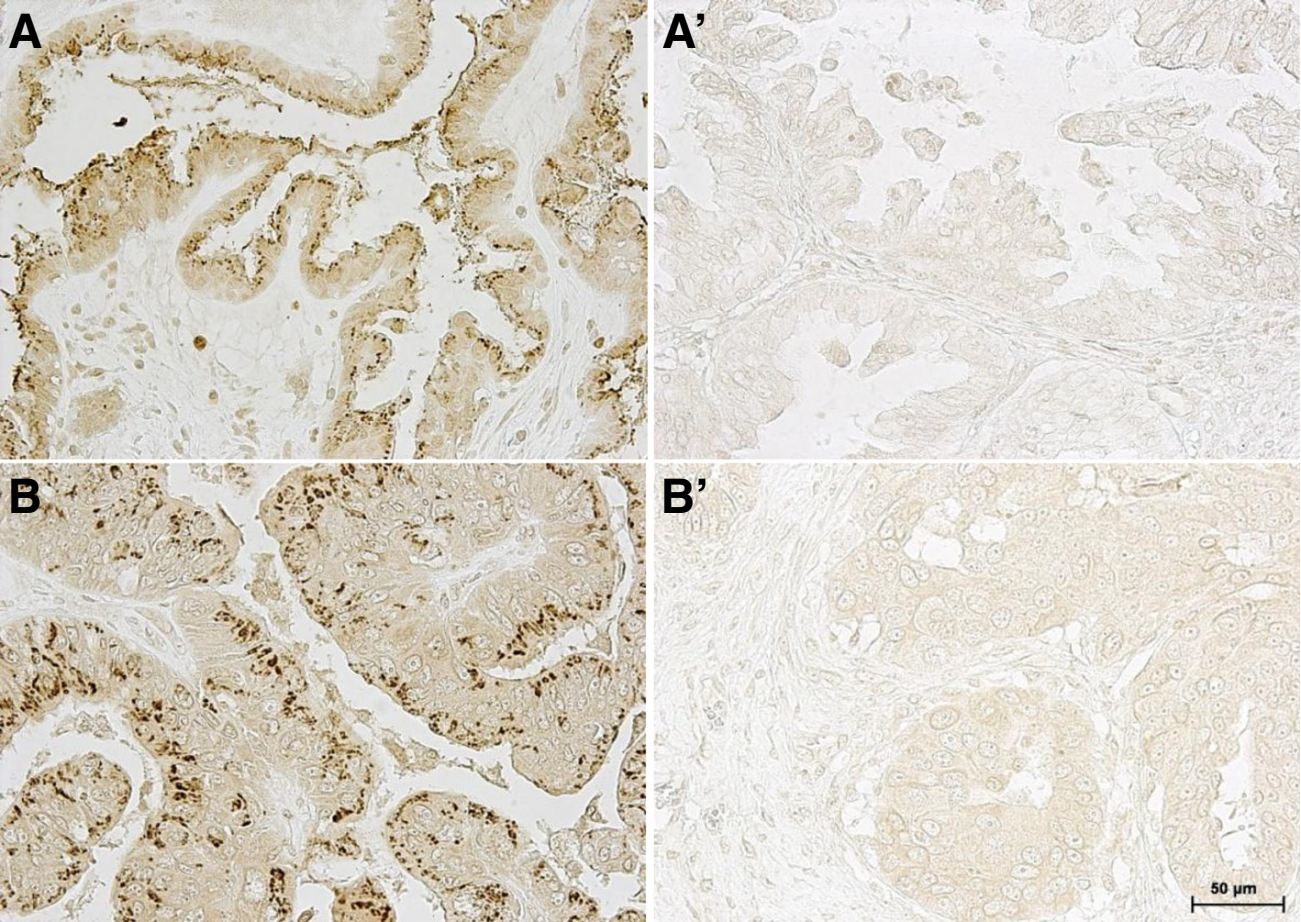
**PRL-3 is overexpressed in human ovarian cancer.** PRL-3 positive signals (brown staining) were mainly detected in the plasma membrane, cytosol, and the Golgi-like sub-cellular structures in the cytoplasm. (**A,A’**) Representative images of PRL-3 overexpression as detected in serous cystadenocarcinoma subtype showing a (**A**) PRL-3 positive and (**A’**) PRL-3 negative sample. (**B,B’**) Representative images of PRL-3 overexpression as detected in endometrioid adenocarcinoma subtype showing a (**B**) PRL-3 positive and (**B’**) PRL-3 negative sample. *Bar*, 50 μm. Magnification, 400X.

Up-regulation of PRL-3 is associated with the metastasis of several types of human cancers [8]. However, evidence suggests that PRL-3 might play an early role in progression of ovarian cancer, prior to metastasis [16]. Using a tissue microarray, we initially screened a total of 175 independent human ovarian cancers and normal tissues using immunohistochemistry to identify the frequency of PRL-3 overexpression. We detected PRL-3 overexpression in 26 out of 135 (19.3%) cancer tissue samples, whereas no PRL-3 expression (0 out of 14) was detected in normal ovarian tissues (Table 1). PRL-3 expression was most closely associated with non-metastatic serous cystadenocarcinoma (29.7% PRL-3 positive) and endometrioid adenocarcinoma (21.7% PRL-3 positive). Representative images of positively- and negatively-stained samples of these 2 subtypes are shown in Figure 1. Strikingly, PRL-3 was absent in all metastatic serous cystadenocarcinoma (LN metastasis) samples analyzed (Table 1). Collectively, these results suggest that PRL-3 is specifically upregulated only in lower grades of ovary cancers, indicating that PRL-3 likely plays an early role in triggering ovarian cancer progression.

**Table 1:** Human ovarian cancer tissue samples staining either positive or negative for PRL-3 expression, as analyzed by immunohistochemistry

**Histo-pathology**	**PRL-3 positive**	**PRL-3 negative**	**% PRL-3 positive**
Normal	0	14	0
Serous cystadenocarcinoma (ovary)	19	64	29.7
Serous cystadenocarcinoma (LN metastasis)	0	10	0
Mucinous cystadenoma	1	18	5.6
Endometriod adenocarcinoma	5	23	21.7
Clear cell carcinoma	0	5	0
Undifferentiated carcinoma	0	3	0
Others *	1	12	8.3
Total	26	149	17.5

*includes tumors of borderline, sarcoma, thecoma, endometrial sinus tumor, granulosa cell tumor, dysgerminoma, uterine tube, tumoral necrosis, and metastatic adenocarcinoma types.

### Knock-down of PRL-3 in A2780 ovarian cancer cells results in reduced migration and invasion

**Figure 2:**
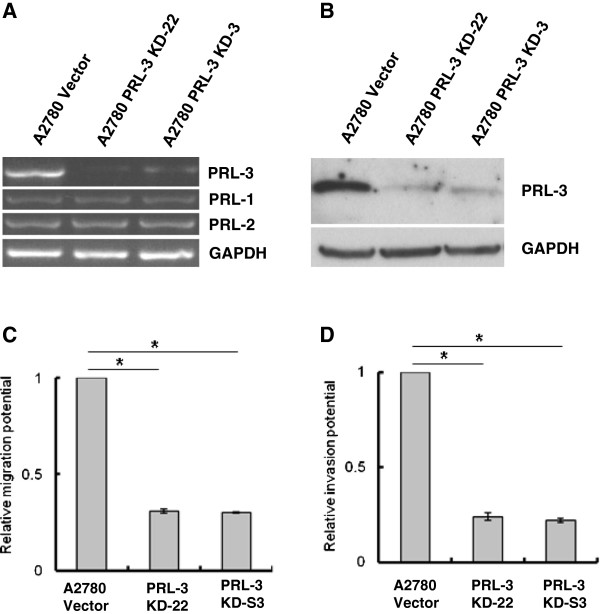
**Knock-down of endogenous PRL-3 inhibits cell migration, invasion, and xenograft tumor growth of A2780 ovarian cancer cells.** (**A**) Human ovarian cancer cells A2780 were transfected with the scrambled control vector or PRL-3 specific shRNA. Stable cell lines (A2780 Vector, A2780 PRL-3 KD-22 and A2780 PRL-3 KD-S3) were harvested and the mRNA levels of PRLs-1, -2, and -3 were analyzed by semi-quantitative RT-PCR using PRL isoform-specific primers. GAPDH mRNA served as a loading control. (**B**) PRL-3 protein levels in A2780 vector, A2780 PRL-3 KD-22 and A2780 PRL-3 KD-S3 were determined by western blot using PRL-3 specific antibody. GADPH was used as a control for the western blot assay. (**C**) Cell migration was analyzed using a standard Transwell assay. After 24 hours incubation, cells that migrated to the lower chamber were fixed, stained, and counted using a light microscope. The relative migration rate of triplicate samples are shown (mean ± SD, Student’s *t*-test, *p < 0.05). (**D**) Matrigel *in vitro* invasion assays were performed as described in the Materials and Methods section. The relative migration rate of triplicate samples are shown (mean ± SD, Student’s *t*-test, *p < 0.05).

To address the function of endogenous PRL-3 in an ovarian cancer model, we transiently depleted A2780 ovarian carcinoma cells, which abundantly express endogenous PRL-3, with various PRL-3 shRNA constructs. After screening 8 unique shRNA constructs for PRL-3 knockdown efficiency (data not shown), stable clones expressing the most two efficiently PRL-3 targeting shRNA (KD-22 and KD-S3) and one scrambled, non-targeting vector control (Vector) were established. A2780 KD-22 and KD-S3 cells displayed efficient and highly selective knockdown of PRL-3, but not closely related family members PRL-1 or PRL-2 (Figure 2A), suggesting that the down-regulation of PRL-3 in KD-22 and KD-S3 cells was specific. The corresponding levels of PRL-3 protein were also reduced in PRL-3 KD-22 and PRL-3 KD-S3 cells compared to vector control cells (Figure 2B). These cell pools were subsequently used for further characterization of PRL-3 function in this study.

To investigate the role of PRL-3 in ovarian cancer cell metastatic processes, cell migration and invasion assays were performed using Transwell migration and Matrigel invasion chambers, respectively. Standard Transwell assays revealed no evident difference in the number of cells moving to the bottom chamber between parental A2780 and scrambled control knockdown cells (data not shown). However, we noted a 70% reduction in PRL-3 KD-22 and PRL-3 KD-S3 cell migration to the bottom chamber 24 h after plating (Figure 2C). Furthermore, we found a 75% reduction in invasive potential of PRL-3 KD-22 and PRL-3 KD-S3 cells compared to control cells (Figure 2D). Collectively, these observations suggest that down-regulation of PRL-3 decreases motility and invasiveness of A2780 ovarian cancer cells.

### Knockdown of PRL-3 results in altered cell morphology

**Figure 3:**
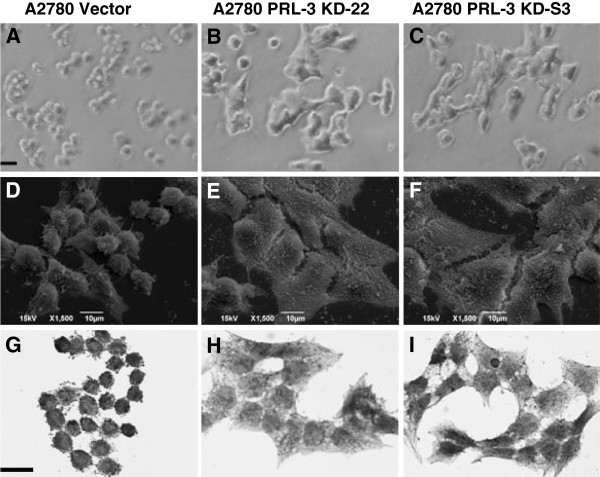
**Knock-down of endogenous PRL-3 leads to altered morphology of A2780 cells.** Representative morphologic micrographs of vector control and A2780 PRL-3 KD cells after 48 h of growth in complete medium are shown. (**A-C**) Light micrographs showing normal morphology of vector control cells versus A2780 PRL-3 KD cells. (**D-F**) Transmission electron micrographs (TEM) showing normal morphology of vector control cells versus the flattened morphology of A2780 PRL-3 KD cells. (**G-I**) Hematoxylin and eosin staining demonstrating control cells versus the spread morphology of A2780 PRL-3 KD cells.

Morphological change plays an important role in many cellular processes such as migration, differentiation and apoptosis. We next investigated whether the decreased motility and invasive ability of PRL-3 KD-22 and PRL-3 KD-S3 cells was coupled to any morphological change. Observation of cells at 50% confluence revealed that down-regulation of PRL-3 induced dramatic changes in cell morphology, as seen using phase-contrast light microscopy (Figure 3A-C). Compared with vector control cells, PRL-3 KD-22 and PRL-3 KD-S3 cells displayed flattened spread morphology and reduced lamellipodia, as examined using electron microscopy (Figure 3D-F). Finally, hematoxylin and eosin staining also showed that PRL-3 knock-down cells spread much wider on glass coverslips than vector control cells (Figure 3G-I).

### PRL-3 downregulates integrin α2 and paxilin expression

**Figure 4:**
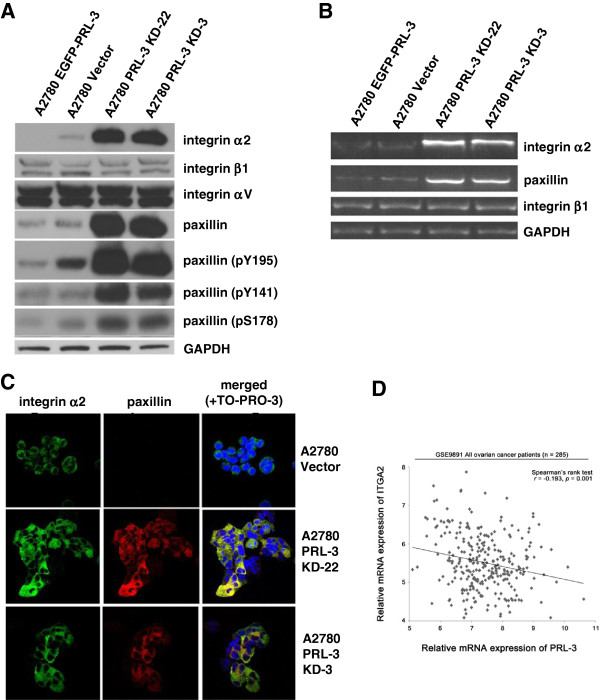
**Knock-down of endogenous PRL-3 expression in A2780 cells upregulates expression of integrin α2 and paxillin.** (**A**) Lysates prepared from the indicated cell lines were examined for various proteins and their phospho-isoforms by immunoblot. GAPDH was used as a loading control. (**B**) Total RNA was extracted from the indicated cell lines and used for RT-PCR assay with the integrin α2-, integrin β1-, or paxilin-specific primer pairs. GAPDH mRNA was used as a loading control. (**C**) Profuse and enhanced expression of integrin α2 (green) and paxillin (red) were detected in A2780 PRL-3 KD cells by indirect immunofluorescence staining. To-pro-3 iodide was used to stain DNA (blue). (**D**) A significant negative correlation between PRL-3 and integrin α2 (ITGA2) mRNA expression levels was observed in primary ovarian cancer specimens from the GSE9891 patient cohort (n = 285, Spearman’s rank test, *r* = −0.193, *p* = 0.001)**.**

To address the possible molecular basis for the altered phenotypes associated with PRL-3 down-regulation, we assessed the expression profiles of various proteins involved in cell adhesion. Of these, we found that PRL-3 knockdown specifically and dramatically enhanced the expression of integrin α2 (Figure 4A). This effect appeared specific, as we noted no changes in expression of integrin β1, its obligatory heterodimer [20], nor any of the other integrins we could detect in our immunoblots (Figure 4A). In addition, no changes were observed in the expression levels of other cell surface adhesion proteins, including the various cadherins (data not shown), suggesting that the regulation of integrin α2 by PRL-3 was highly specific. Paxillin, a key signaling protein downstream of integrin, was similarly found to be dramatically enhanced, both in expression and phosphorylation on Y195, Y141 and S178, in PRL-3-ablated cells (Figure 4A). Semi-quantitative RT-PCR assays indicated that enhanced RNA levels also contributed to the increased protein levels of both integrin α2 and paxillin (Figure 4B). Importantly, overexpression of EGFP-PRL-3 reduced both the RNA and protein levels of integrin α2 and paxillin (Figure 4A, B), suggesting that the regulation of these proteins was both specific and sensitive to PRL-3 expression levels. Immunofluorescence analysis further verified the upregulation of both integrin α2 and paxillin in PRL-3-ablated cells (Figure 4C). Interestingly, PRL-3 knockdown did not influence FAK expression, but slightly enhanced FAK phosphorylation (Additional file 2: Figure S1). Thus, the downregulation of PRL-3 in A2780 releases suppression of integrin α2 and paxilin expression, resulting in the robust increase of these 2 key adhesion proteins at both mRNA and protein levels.

To investigate the clinical relevance of our observations, we analyzed a microarray dataset comprising 285 primary ovarian cancer patient specimens [19]. We found a significant negative correlation between PRL-3 and integrin α2 (ITGA2) mRNA expression levels (Spearman’s rank test, r = −0.193, p = 0.001; Figure 4D). This finding corroborates our *in vitro* observations and further suggests a clinical relevance for PRL-3 suppression of integrin α2 expression in ovarian cancer.

### PRL-3 depletion results in upregulation of c-fos expression

**Figure 5:**
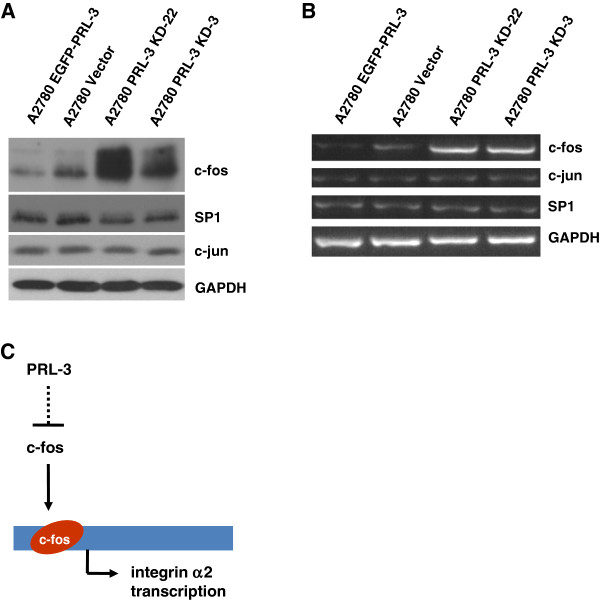
**Up-regulation of c-fos expression in PRL-3 depleted cells.** (**A**) Cell lysates prepared from the indicated cell lines were examined for the protein levels of c-fos, c-jun and Sp1 by immunoblot. GAPDH was used as a loading control. (**B**) Total RNA extracted from the indicated cell lines was used for semi-quantitative RT-PCR assay with c-fos-, c-jun- and Sp1-specific primer pairs. GAPDH was used as a control. (**C**) Model of PRL-3 mediated regulation of integrin α2 via c-Fos.

Because PRL-3 ablation enhanced both RNA and protein levels of integrin α2, we next investigated the levels of c-fos, c-jun and Sp1, which have previously been identified as transcription factors regulating integrin α2 expression [21,22]. We found the protein levels of c-fos, but not Sp1 and c-jun, to be markedly enhanced in PRL-3 knockdown cells (Figure 5A). In addition, using semi-quantitative RT-PCR, we found that c-fos RNA levels were increased markedly in PRL-3 knockdown cells compared with the control, while RNA levels of Sp1 and c-jun did not show evident changes (Figure 5B). In agreement with the knockdown data, semi-quantitative RT-PCR and western blot assays indicated that overexpression of EGFP-PRL-3 in turn reduced the RNA and protein levels of c-fos in A2780 cells (Figure 5A, B). Collectively, the data suggests that PRL-3 might inhibit c-fos expression as a means of suppressing integrin α2 expression. A model describing the relationship between PRL-3, c-fos and integrin α2 in promoting ovarian cancer progression is hereby proposed (Figure 5C).

### Knockdown of PRL-3 in A2780 reduces tumorigenicity *in vivo*

**Figure 6:**
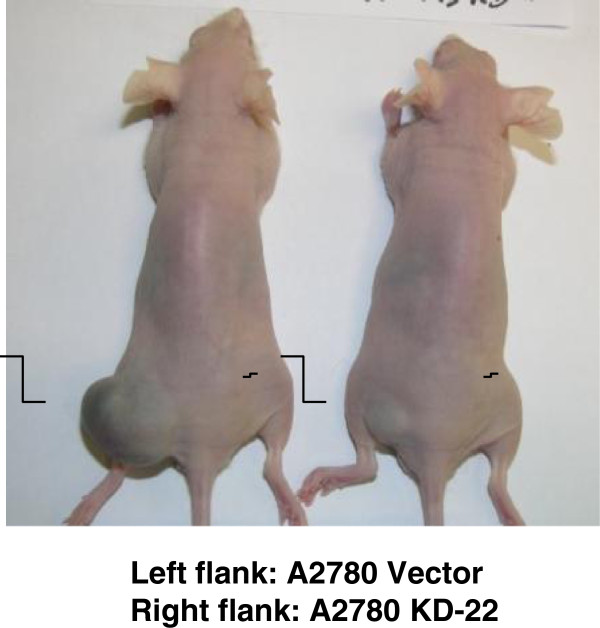
**Depletion of PRL-3 expression abolishes the tumorigenic potential of A2780 cells*in vivo*.** 1 × 10^6^ A2780 control or PRL-3 KD-22 cells were injected into the left and right hind flanks, respectively, of nude mice and allowed to grow for up to 5 weeks. Representative results of tumor formation are shown. *Arrows*, tumors formed by A2780 Vector (control) cells; *arrowheads*, tumors formed by A2780 PRL-3 KD-22 cells.

To directly examine the function of PRL-3 in tumorigenesis *in vivo*, we injected A2780 vector control and A2780 PRL-3 KD cells into the hip areas of nude mice and monitored tumor growth for up to 5 weeks. Compared to A2780 Vector cells which formed large tumors (Figure 6, left hips, arrows), A2780 PRL-3 KD cells failed to form tumors *in vivo* (Figure 6, right hips, arrowheads). Since the knock-down of PRL-3 abolishes tumorigenic potential of A2780 cells, these results suggest that PRL-3 acts as a critical tumor promoter for A2780 cells *in vivo.*

## Discussion and conclusion

Accumulating evidence indicates that the dysregulated expression of PRLs are linked to the genesis and progression of human cancers, suggesting the PRL-PTP family as emerging biomarkers for cancer prognosis and promising therapeutic targets [8,10]. Here, we present evidence that PRL-3 is specifically upregulated in low-grade human ovarian cancers, particularly the serous cystadenocarcinoma and endometriod adenocarcinoma subtypes, but is undetectable in normal ovarian tissues. Depletion of PRL-3 resulted in reduced invasion, motility, and tumorigenic potential of A2780 ovarian cancer cells. We further provide evidence for the PRL-3-mediated suppression of integrin α2 and paxillin, 2 key cell adhesion proteins, in A2780 ovarian carcinoma cells. c-fos, an integrin α2 transcriptional regulator, was also identified as a tightly suppressed protein by PRL-3. Importantly, we noted a significant negative correlation between PRL-3 and integrin α2 in human ovarian cancer specimens. Collectively, our results suggest that PRL-3 plays multiple roles in early progression of human ovarian cancer.

In this study, we showed that elevated PRL-3 expression associated closely with 2 subtypes of ovarian cancers – serous cystadenocarcinomas and endometroid adenocarcinomas. Notably, ovarian serous cystadenocarcinoma is the most common subtype of epithelial ovarian cancer, accounting for almost 90% of all ovarian cancers [23]. The high frequency of PRL-3 expression observed in this subtype suggests that PRL-3 might play important role in the majority of ovarian cancer patients which may have higher risk in developing more advanced cancer metastasis. Interestingly, we failed to note any elevated PRL-3 expression in the lymph node metastasis samples from primary serous cystadenocarcinomas, an observation in line with a previous report suggesting an early role of PRL-3 in ovarian cancer progression [16]. Given the high frequency of the serous cystadenocarcinoma subtype of ovarian cancer, we envision a significant value of PRL-3 as an early prognostic marker in clinical diagnosis for such patients to receive early attention for cancer intervention. Importantly, in light of our recent results demonstrating the value of anti-PRL-3 antibody therapy against A2780 ovarian cancer metastatic tumors [24-26], we hereby propose anti-PRL-3 therapy as a viable approach to treat PRL-3-positive ovarian cancer patients as well.

Intriguingly, besides growth and invasion defects, marked morphologic changes were observed for the established PRL-3 knockdown cells. PRL-3 deleted cells were flatter and spread wider on culture plates compared with parental cells. It is well-accepted that when cells move or undergo morphologic changes, the expression of adhesion molecules, especially integrin subunits, are dynamically regulated. Notably, the turnover of cell-matrix adhesions is always accompanied by alterations in cell morphology and invasive ability [3]. Here, we noted morphologic changes induced by PRL-3 depletion which corresponded to a dramatic increase in expression of integrin α2. Integrin α2 has been reported to play a role in suppressing pancreatic cancer invasion [27]. Previously, it was shown in breast carcinoma cells that decreasing the expression of α2β1 integrin resulted in dramatic morphological alterations, while re-expression of α2β1 integrin restored the ability to differentiate and markedly reduced *in vivo* tumorigenicity [6]. Recently, PRL-3 was shown to directly interact and regulate the activity of integrin β1 in an integrin α1-dependent manner [28]. Interestingly, integrin β1 is a heterodimeric partner for both integrin α1 and α2 [29]. Among the eight integrin family members examined in this study (α2, α5, αV, β1; integrins α3, α4, β3, and β4 were undetectable in immunoblots), only the expression levels of integrin α2 were found to tightly correlate with PRL-3 expression (Figure 4A; data not shown), suggesting that PRL-3 may specifically regulate integrin α2 in human ovarian cancer. It should be noted that our study did not investigate integrin activation status, which may reveal additional regulation of integrins by PRL-3. Nonetheless, in light of the recent finding that integrin α2β1 heterodimer is a metastasis suppressor of murine and human cancers [7], one could envisage PRL-3 to potently promote cancer progression towards metastatic dissemination by concurrently downregulating both the expression of integrin α2 and the activation of integrin β1. Taken with our results here, PRL-3-mediated suppression of integrin α2 likely further contributes to PRL-3’s role in promoting ovarian cancer motility, invasiveness and tumorigenicity.

In summary, we showed dramatic morphologic changes associated with inhibited cell motility and invasion in PRL-3-ablated ovarian cancer cells. Our results suggest a plausible involvement of c-fos, and consequently integrin α2, in PRL-3-mediated cell adhesion and migration processes. The links between PRL-3 and c-fos have yet to be addressed. Due to a repertoire of transcriptional response elements in the *cfos* promoter, c-fos is regulated in response to diverse extracellular signals [30]. Indeed, the *in vivo* transcriptional regulation of *cfos* could only be faithfully mimicked by a reporter controlled by the whole intact gene sequence [31]. This suggests that higher order complexes involving specific transcription activators and coactivators integrate diverse signals to elaborate a controlled response. To this end, the precise mechanism of PRL-3 mediated c-fos upregulation is a subject of ongoing studies. FAK and paxillin are recruited to intracellular tails of integrin and mediate several downstream responses, including cell migration [4]. Phosphorylation of FAK and paxillin are involved in their kinase activity and protein binding ability, respectively [32]. Since integrins regulate the association and phosphorylation of paxillin [33], the profuse phosphorylation of paxillin, and to a lower extent FAK, suggests hyperactive signaling induction in PRL-3-ablated cells. Although more work will be needed to address the contribution of c-fos and integrin α2 to ovarian cancer progression, our study highlights the importance of PRL-3 as a potential early biomarker and therapeutic target in human ovarian cancers.

## Competing interests

The authors declare that they have no competing interests.

## Authors’ contributions

LH and ZQ designed research; LH, WH, GK, LJ and ZH performed research; LH, AQQ and ZQ analyzed data, LH, AQQ and ZQ wrote the paper. All authors read and approved the final manuscript.

## Pre-publication history

The pre-publication history for this paper can be accessed here:

http://www.biomedcentral.com/1471-2407/13/80/prepub

## Supplementary Material

Additional file 1: Table S1 Primer sequences used for semi-quantitative RT-PCR. Click here for file

Additional file 2: Figure S1 Lysates prepared from the indicated cell lines were examined for FAK and its phospho-isoforms by immunoblot. GAPDH was used as a loading control. Click here for file

## References

[B1] Fidler IJ The pathogenesis of cancer metastasis: the ‘seed and soil’ hypothesis revisited Nat Rev Cancer 2003 3 453 58 10.1038/nrc1098 12778135

[B2] Hanahan D Weinberg RA The hallmarks of cancer Cell 2000 100 57 70 10.1016/S0092-8674(00)81683-9 10647931

[B3] Juliano RL Varner JA Adhesion molecules in cancer: the role of integrins Curr Opin Cell Biol 1993 5 812 18 10.1016/0955-0674(93)90030-T 8240825

[B4] Truong H Danen EHJ Integrin switching modulates adhesion dynamics and cell migration Cell adhesion & migration 2009 3 179 81 10.4161/cam.3.2.8036 19287215 PMC2679881

[B5] Chang YS Chen YQ Timar J Nelson KK Grossi IM Fitzgerald LA Diglio CA Honn KV Increased expression of alpha IIb beta 3 integrin in subpopulations of murine melanoma cells with high lung-colonizing ability Int J Cancer 1992 51 445 51 10.1002/ijc.2910510318 1375589

[B6] Zutter MM Santoro SA Staatz WD Tsung YL Re-expression of the alpha 2 beta 1 integrin abrogates the malignant phenotype of breast carcinoma cells Proc Natl Acad Sci U S A 1995 92 7411 15 10.1073/pnas.92.16.7411 7638207 PMC41349

[B7] Ramirez NE Zhang Z Madamanchi A Boyd KL O’Rear LD Nashabi A Li Z Dupont WD Zijlstra A Zutter MM The α2β1 integrin is a metastasis suppressor in mouse models and human cancer J Clin Invest 2011 121 226 37 10.1172/JCI42328 21135504 PMC3007139

[B8] Al-Aidaroos AQO Zeng Q PRL-3 phosphatase and cancer metastasis J Cell Biochem 2010 111 1087 98 10.1002/jcb.22913 21053359

[B9] Saha S Bardelli A Buckhaults P Velculescu VE Rago C St Croix B Romans KE Choti MA Lengauer C Kinzler KW Vogelstein B A phosphatase associated with metastasis of colorectal cancer Science 2001 294 1343 46 10.1126/science.1065817 11598267

[B10] Bessette DC Qiu D Pallen CJ PRL PTPs: mediators and markers of cancer progression Cancer Metastasis Rev 2008 27 231 52 10.1007/s10555-008-9121-3 18224294

[B11] Zeng Q Dong JM Guo K Li J Tan HX Koh V Pallen CJ Manser E Hong W PRL-3 and PRL-1 promote cell migration, invasion, and metastasis Cancer Res 2003 63 2716 22 12782572

[B12] Kato H Semba S Miskad UA Seo Y Kasuga M Yokozaki H High expression of PRL-3 promotes cancer cell motility and liver metastasis in human colorectal cancer: a predictive molecular marker of metachronous liver and lung metastases Clin Cancer Res 2004 10 7318 28 10.1158/1078-0432.CCR-04-0485 15534108

[B13] Rouleau C Roy A St Martin T Dufault MR Boutin P Liu D Zhang M Puorro-Radzwill K Rulli L Reczek D Bagley R Byrne A Weber W Roberts B Klinger K Brondyk W Nacht M Madden S Burrier R Shankara S Teicher BA Protein tyrosine phosphatase PRL-3 in malignant cells and endothelial cells: expression and function Mol Cancer Ther 2006 5 219 29 10.1158/1535-7163.MCT-05-0289 16505094

[B14] Qian F Li YP Sheng X Zhang ZC Song R Dong W Cao SX Hua ZC Xu Q PRL-3 siRNA inhibits the metastasis of B16-BL6 mouse melanoma cells in vitro and in vivo Mol Med 2007 13 151 59 17592549 10.2119/2006-00076.Qian PMC1892759

[B15] Cai SR Wang Z Chen CQ Wu WH He YL Zhan WH Zhang CH Cui J Wu H Role of silencing phosphatase of regenerationg liver-3 expression by microRNA interference in the growth of gastric cancer Chin Med J (Engl) 2008 121 2534 38 19187591

[B16] Polato F Codegoni A Fruscio R Perego P Mangioni C Saha S Bardelli A Broggini M PRL-3 phosphatase is implicated in ovarian cancer growth Clin Cancer Res 2005 11 6835 39 10.1158/1078-0432.CCR-04-2357 16203771

[B17] Wang H Quah SY Dong JM Manser E Tang JP Zeng Q PRL-3 down-regulates PTEN expression and signals through PI3K to promote epithelial-mesenchymal transition Cancer Res 2007 67 2922 26 10.1158/0008-5472.CAN-06-3598 17409395

[B18] Li J Guo K Koh VWC Tang JP Gan BQ Shi H Li HX Zeng Q Generation of PRL-3- and PRL-1-specific monoclonal antibodies as potential diagnostic markers for cancer metastases Clin Cancer Res 2005 11 2195 204 10.1158/1078-0432.CCR-04-1984 15788667

[B19] Tothill RW Tinker AV George J Brown R Fox SB Lade S Johnson DS Trivett MK Etemadmoghadam D Locandro B Traficante N Fereday S Hung JA Chiew YE Haviv I Australian Ovarian Cancer Study Group, Gertig D, DeFazio A, Bowtell DDL: Novel molecular subtypes of serous and endometrioid ovarian cancer linked to clinical outcome Clin Cancer Res 2008 14 5198 208 10.1158/1078-0432.CCR-08-0196 18698038

[B20] Giancotti FG Ruoslahti E Integrin signaling Science 1999 285 1028 32 10.1126/science.285.5430.1028 10446041

[B21] Zutter MM Ryan EE Painter AD Binding of phosphorylated Sp1 protein to tandem Sp1 binding sites regulates alpha2 integrin gene core promoter activity Blood 1997 90 678 89 9226168

[B22] Zutter MM Painter AD Yang X The Megakaryocyte/Platelet-specific enhancer of the alpha2beta1 integrin gene: two tandem AP1 sites and the mitogen-activated protein kinase signaling cascade Blood 1999 93 1600 11 10029589

[B23] Kaplan B Markman M Eifel P DeVita VJ, Hellman S, Rosenberg S Ovarian Cancer, Peritoneal Carcinoma and Fallopian Tube Carcinoma Cancer: Principles and Practice of Oncology 2005 7 Philadelphia: Lippincott Williams & Wilkins 1364

[B24] Guo K Tang JP Tan CPB Wang H Zeng Q Monoclonal antibodies target intracellular PRL phosphatases to inhibit cancer metastases in mice Cancer Biol Ther 2008 7 750 57 10.4161/cbt.7.5.5764 18364570

[B25] Guo K Tang JP Li J Al-Aidaroos AQO Hong CW Tan CPB Park JE Varghese L Feng Z Zhou J Chng WJ Zeng Q Engineering the first chimeric antibody in targeting intracellular PRL-3 oncoprotein for cancer therapy in mice Oncotarget 2012 3 158 71 22374986 10.18632/oncotarget.442 PMC3326646

[B26] Hong CW Zeng Q Awaiting a new era of cancer immunotherapy Cancer Res 2012 72 3715 19 10.1158/0008-5472.CAN-12-0063 22815525

[B27] Lee CY Marzan D Lin G Goodison S Silletti S α2 Integrin-Dependent Suppression of Pancreatic Adenocarcinoma Cell Invasion Involves Ectodomain Regulation of Kallikrein-Related Peptidase-5 Journal of oncology 2011 2011 365651 22203845 10.1155/2011/365651 PMC3245846

[B28] Tian W Qu L Meng L Liu C Wu J Shou C Phosphatase of regenerating liver-3 directly interacts with integrin beta1 and regulates its phosphorylation at tyrosine 783 BMC Biochem 2012 13 22 10.1186/1471-2091-13-22 23092334 PMC3558359

[B29] Varner JA Cheresh DA Integrins and cancer Curr Opin Cell Biol 1996 8 724 30 10.1016/S0955-0674(96)80115-3 8939661

[B30] Coulon V Chebli K Cavelier P Blanchard JM A novel mouse c-fos intronic promoter that responds to CREB and AP-1 is developmentally regulated in vivo PloS one 2010 5 e11235 10.1371/journal.pone.0011235 20574536 PMC2888593

[B31] Robertson LM Kerppola TK Vendrell M Luk D Smeyne RJ Bocchiaro C Morgan JI Curran T Regulation of c-fos expression in transgenic mice requires multiple interdependent transcription control elements Neuron 1995 14 241 52 10.1016/0896-6273(95)90282-1 7857636

[B32] Barczyk M Carracedo S Gullberg D Integrins. Cell Tissue Res 2010 339 269 80 10.1007/s00441-009-0834-6 PMC2784866 19693543

[B33] Lewis JM Schwartz MA Integrins regulate the association and phosphorylation of paxillin by c-Abl J Biol Chem 1998 273 14225 30 10.1074/jbc.273.23.14225 9603926

