# Supplementary material for: PRL-3 suppresses c-Fos and integrin α2 expression in ovarian cancer cells
Source: BMC Cancer. 2013 Feb 18;13:80. doi: 10.1186/1471-2407-13-80 (PMC3620920; doi:10.1186/1471-2407-13-80)
Supplement: Additional file 1: Table S1 — Primer sequences used for semi-quantitative RT-PCR. [file 1471-2407-13-80-S1.ppt]

## Slide 1
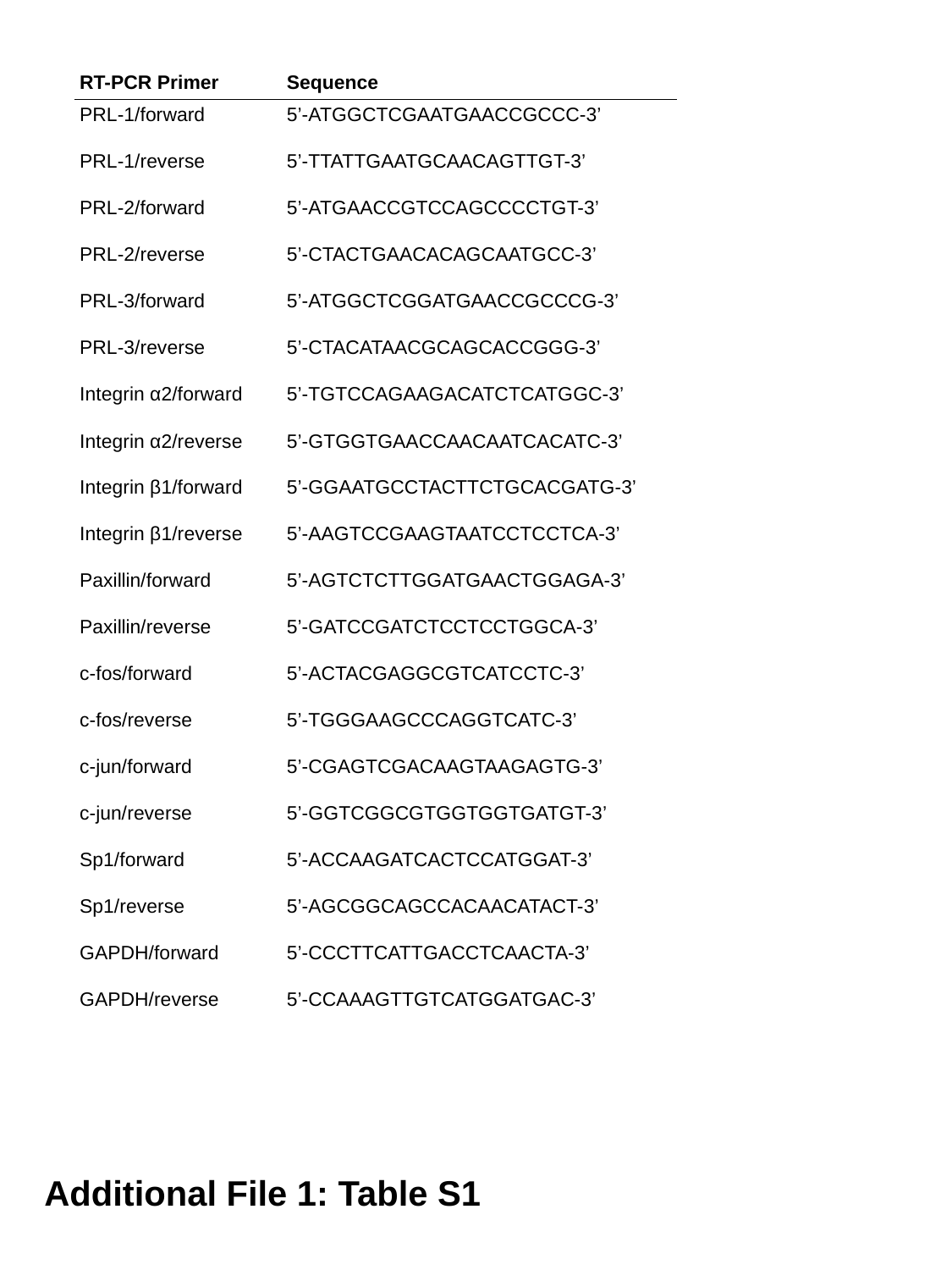

| RT-PCR Primer | Sequence |
| --- | --- |
| PRL-1/forward | 5’-ATGGCTCGAATGAACCGCCC-3’ |
| PRL-1/reverse | 5’-TTATTGAATGCAACAGTTGT-3’ |
| PRL-2/forward | 5’-ATGAACCGTCCAGCCCCTGT-3’ |
| PRL-2/reverse | 5’-CTACTGAACACAGCAATGCC-3’ |
| PRL-3/forward | 5’-ATGGCTCGGATGAACCGCCCG-3’ |
| PRL-3/reverse | 5’-CTACATAACGCAGCACCGGG-3’ |
| Integrin α2/forward | 5’-TGTCCAGAAGACATCTCATGGC-3’ |
| Integrin α2/reverse | 5’-GTGGTGAACCAACAATCACATC-3’ |
| Integrin β1/forward | 5’-GGAATGCCTACTTCTGCACGATG-3’ |
| Integrin β1/reverse | 5’-AAGTCCGAAGTAATCCTCCTCA-3’ |
| Paxillin/forward | 5’-AGTCTCTTGGATGAACTGGAGA-3’ |
| Paxillin/reverse | 5’-GATCCGATCTCCTCCTGGCA-3’ |
| c-fos/forward | 5’-ACTACGAGGCGTCATCCTC-3’ |
| c-fos/reverse | 5’-TGGGAAGCCCAGGTCATC-3’ |
| c-jun/forward | 5’-CGAGTCGACAAGTAAGAGTG-3’ |
| c-jun/reverse | 5’-GGTCGGCGTGGTGGTGATGT-3’ |
| Sp1/forward | 5’-ACCAAGATCACTCCATGGAT-3’ |
| Sp1/reverse | 5’-AGCGGCAGCCACAACATACT-3’ |
| GAPDH/forward | 5’-CCCTTCATTGACCTCAACTA-3’ |
| GAPDH/reverse | 5’-CCAAAGTTGTCATGGATGAC-3’ |
Additional File 1: Table S1
