# Supplementary figures and images for: PRL-3 suppresses c-Fos and integrin α2 expression in ovarian cancer cells
Source: BMC Cancer. 2013 Feb 18;13:80. doi: 10.1186/1471-2407-13-80 (PMC3620920; doi:10.1186/1471-2407-13-80)

## Slide 1
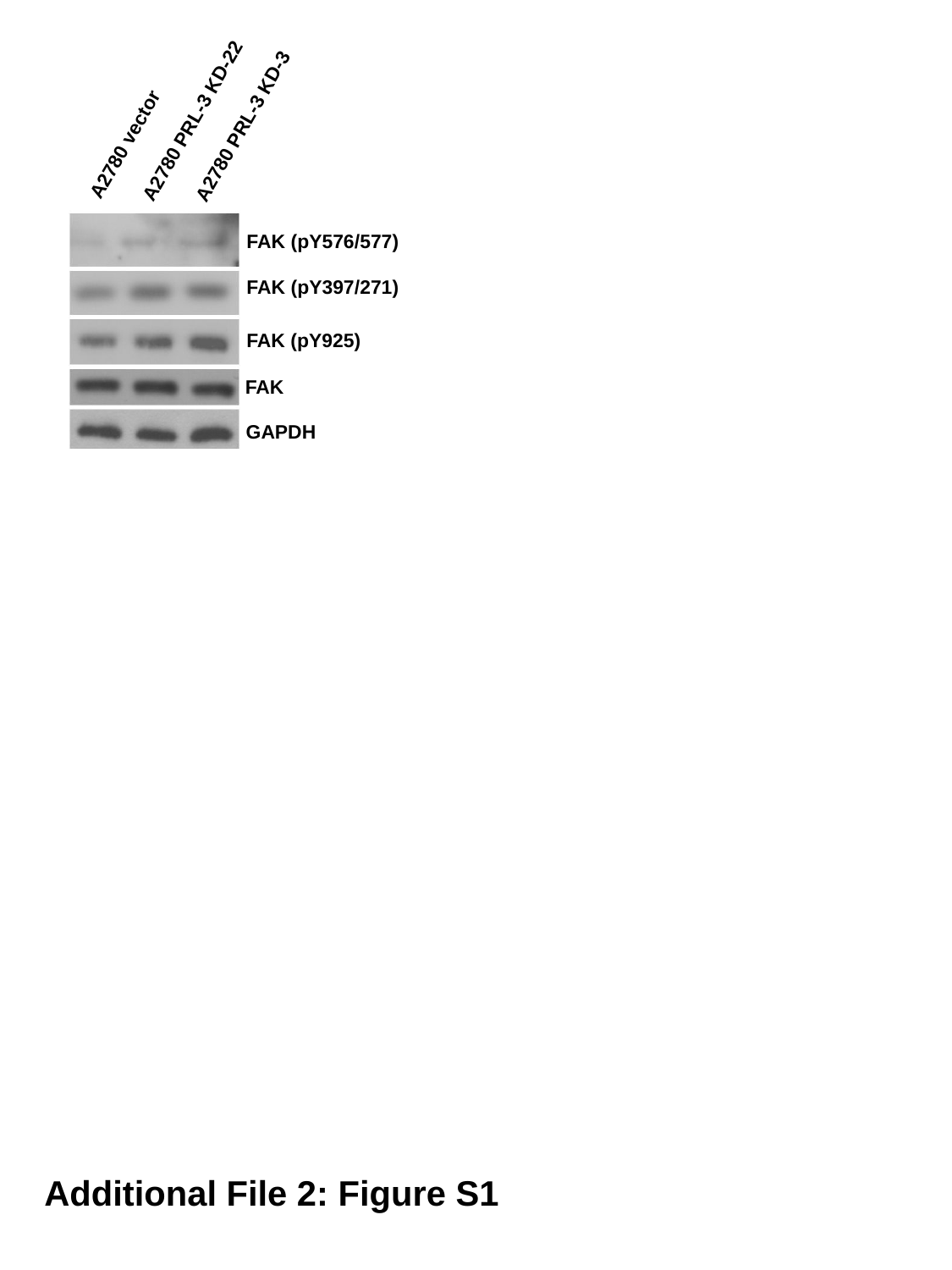

A2780 PRL-3 KD-22
A2780 PRL-3 KD-3
A2780 vector
FAK (pY576/577)
FAK (pY397/271)
FAK (pY925)
FAK
GAPDH
Additional File 2: Figure S1

Supplement: Additional file 2: Figure S1 — Lysates prepared from the indicated cell lines were examined for FAK and its phospho-isoforms by immunoblot. GAPDH was used as a loading control. [file 1471-2407-13-80-S2.ppt]
